# Performance Analysis of Direct GPS Spoofing Detection Method with AHRS/Accelerometer

**DOI:** 10.3390/s20040954

**Published:** 2020-02-11

**Authors:** Keum-Cheol Kwon, Duk-Sun Shim

**Affiliations:** School of Electrical and Electronics Engineering, Chung-Ang University, 84 Huksuk-ro, Dongjak-ku, Seoul 06974, Korea; kckwon@cau.ac.kr

**Keywords:** GPS, spoofing detection, AHRS, accelerometer, Shim probability density function

## Abstract

The global positioning system (GPS) is an essential technology that provides positioning capabilities and is used in various applications such as navigation, surveying, mapping, robot simultaneous localization and mapping (SLAM), location-based service (LBS), etc. However, the GPS is known to be vulnerable to intentional attacks such as spoofing because of its simple signal structure. In this study, a direct method is proposed for GPS spoofing detection, using Attitude and Heading Reference System (AHRS) accelerometer and analyzing the detection performance with corresponding probability density functions (PDFs). The difference in the acceleration between the GPS receiver and the accelerometer is used to detect spoofing. The magnitude of the acceleration error may be used as a decision variable. Additionally, using the magnitude of the north (or east) component of the acceleration error as another decision variable is proposed, which shows better performance in some conditions. The performance of the two decision variables is compared by calculating the probability of spoofing detection and the detectable minimum spoofing acceleration (DMSA), given a pre-defined false alarm probability and a pre-defined detection probability. It turns out that both decision variables need to be used together to obtain the best spoofing detection performance.

## 1. Introduction

The global navigation satellite system (GNSS) is an essential technology for positioning and timing, and its application covers various areas such as navigation, surveying, mapping, robot simultaneous localization and mapping (SLAM), location-based service (LBS), etc. The global positioning system (GPS) is the original GNSS and its full operational capability (FOC) was declared in 1995 in the United States of America. The legacy L1 C/A code signal of GPS is very weak at the Earth’s surface and has a simple structure to implement [1,2]. Thus, the GPS signal is vulnerable to intentional interferences such as jamming and spoofing. While jamming attacks make the GPS receivers malfunction, spoofing attacks make the target receiver unaware of it being attacked by the spoofer. Spoofing threats have garnered attention since the initial finding of the 2001 Volpe Report [3]. GPS spoofers may cause significant damage to the target receiver by transmitting counterfeit navigation data which can result in erroneous navigation. Thus, spoofing attacks are a significant problem to users and many studies on spoofing attacks and anti-spoofing methods have been carried out since 2001.

Experiments have been conducted to understand the vulnerability of the GPS to spoofing [4,5], which proved that the GPS is not secure from spoofing. Some experiments tried to implement the spoofer itself [5,6,7]. A variety of approaches have been studied about spoofing detection of the GNSS [7,8,9,10,11,12,13,14,15,16,17,18] such as using received signal strength [9], signal quality monitoring at code and carrier levels [7], phase-only analysis of variance (PANOVA) method with generalized likelihood ratio test (GLRT) by employing dual antenna system [10], and using maximum likelihood estimator (MLE) [11]. Prior studies [7,12] summarize various spoofing detection techniques, among which low complexity spoofing detection techniques include $C/N_{0}$ monitoring, absolute power monitoring, power variation versus receiver movement, code and phase rate consistency check, and GPS clock consistency check, while high complexity spoofing detection techniques include Direction Of Arrival (DOA) comparison [14] and consistency check with other solutions [15,16,17,18].

Among the effective high complexity spoofing detection techniques mentioned in the prior study [12], the DOA comparison approach uses the DOA measurements to assess the direction of the spoofing source and mitigates the interference by placing the spatial null in the antenna reception pattern [14]. The consistency check approach uses the inertial measurement unit (IMU) [15,16,17,18]. The relative platform trajectory estimated by the GNSS receiver is compared to the relative trajectory developed from the IMU measurement, specifically gyro output, to detect spoofing [15]. In References [16,17], a tightly coupled GPS / inertial navigation system (INS) is used to detect spoofing by incorporating fault detection concepts and Kalman filter, respectively, based on receiver autonomous integrity monitoring (RAIM). In [18], an accelerometer is used to detect spoofing by comparison of acceleration between the GPS receiver and the IMU. However, no prior research has been done on using the IMU and the acceleration error which is expressed with exact probability density function.

In this study, a direct GPS spoofing detection method is proposed which uses attitude and heading reference system (AHRS) and accelerometers via direct comparison of the acceleration estimated from the GPS receiver and the acceleration measured from IMU, which provides the acceleration error. The acceleration from the GPS receiver is estimated from the Kalman filter by including the acceleration as a state variable of the GPS dynamic system in this study, while the acceleration from the GPS receiver in Reference [18] is obtained from the difference of velocities which are estimated from the GPS Kalman filter. Two decision variables for spoofing detection are defined: the acceleration error from the acceleration of the GPS receiver and the acceleration from the AHRS/Accelerometer, expressed in the navigation frame. One decision variable is defined as the magnitude of the acceleration error, where only horizontal component is used, and the probability density function (PDF) of the decision variable is derived. It is called Shim PDF. The other decision variable is the magnitude of the north (or east) component of the acceleration error. The PDF of this decision variable is the folded Gaussian PDF. It was found that in the special condition that both moving acceleration and spoofing acceleration head around north (or east) together, the second decision variable of the magnitude of the north (or east) component provides better detection performance than the first decision variable of the magnitude of the horizontal component. Thus the magnitude of the horizontal acceleration error, the magnitude of the north component of the acceleration error, and the magnitude of the east component of the acceleration error are proposed to be used together to check whether one of these is greater than or equal to the corresponding threshold to detect the GPS spoofing.

Two performance measures are considered for spoofing detection. One is the probability of spoofing detection. The exact PDF for both decision variables is known and thus, for a given probability of false alarm, the detection threshold and the detection probability can be calculated. The other is the detectable minimum spoofing acceleration (DMSA), which is the magnitude of the minimum spoofing acceleration to obtain a pre-defined detection probability, given a pre-defined false alarm probability. The performances of the two decision variables for the two performance measures are compared and analyzed.

The structure of the first-half of the proposed direct GPS spoofing detection method is given in Section 2, which provides the acceleration error with GPS Kalman filter and AHRS. Section 3 defines the acceleration error, and two decision variables with their probability density functions, and shows the second-half of the proposed direct GPS spoofing detection method. The performance analysis of the proposed direct GPS spoofing detection method is given for two decision variables in Section 4 and Section 5 using the two performance measures, the probability of detection and the DMSA. The conclusion is presented in Section 6.

## 2. The Structure of the First-half of the Proposed Direct GPS Spoofing Detection Method

### 2.1. Block Diagram to Obtain the Acceleration Error from GPS Receiver and Accelerometer

**Figure 1:**
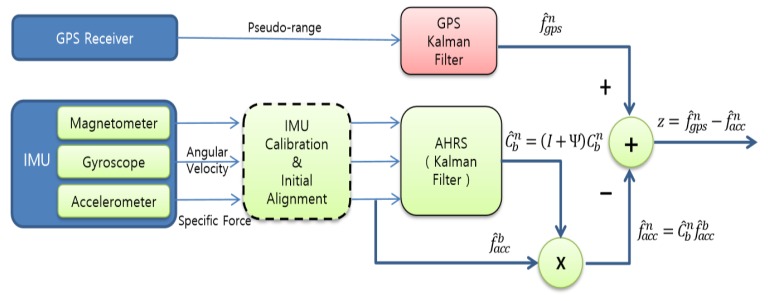
Block diagram to obtain the acceleration error from the Global Positioning System (GPS) receiver and accelerometer.

In this study, GPS spoofing detection is done by using the comparison of accelerations obtained from the GPS receiver and accelerometers. The block diagram of Figure 1 shows the procedure to obtain the difference of the two acceleration measurements. The accelerometers measure the specific force ${\hat{f}}_{acc}^{b}$ and then, it is changed into ${\hat{f}}_{acc}^{n}$ through the transformation matrix $C_{b}^{n}$. In ${\hat{f}}_{acc}^{b}$ and ${\hat{f}}_{acc}^{n}$, the superscript *b* denotes the body frame and *n* denotes the navigation frame. The navigation frame uses the north(x)-east(y)-down(z) (NED) frame in this study. AHRS produces the transformation matrix$\;C_{b}^{n}$ by using the sensor outputs and Kalman filter. $C_{b}^{n}$ denotes the direction cosine matrix from the body frame to the navigation frame. The hat (^) denotes measured or calculated values containing errors and Ψ denotes the skew symmetric matrix of the attitude error.

It is assumed that IMU calibration and initial alignment is performed in advance depending on the characteristics of various inertial sensors since there are many kinds of gyroscopes, such as ring laser gyro, fiber optic gyro, hemispherical resonator gyro, and low cost micro-electro-mechanical system (MEMS) gyro, and so on, and different gyroscope has different error sources, and accelerometer has also many types, such as pendulous type, vibrating type, silicon type, and MEMS type. Figure 1 shows the IMU calibration and initial alignment with the dotted block, which will not be considered in this paper. Thus misalignment, bias, scale factor, and others are assumed to be compensated in advance. The IMU calibration and initial alignment is an essential process in the inertial navigation system and thus there are much research results which have been already performed [19,20]. Only GPS spoofing detection will be considered in this paper.

Matrices use capital letters, and vectors, small letters. Matrices and vectors will use bold letters and scalars use plain letters.

### 2.2. GPS Kalman Filter

The GPS receiver usually provides position and velocity information. To obtain acceleration from the GPS receiver, the Kalman filter is used by including acceleration as a state variable. The dynamics of the GPS receiver can be described by the state-space model as in Equation (1), which has 11 state variables such as 3-dimensional position, velocity, acceleration, GPS receiver’s clock bias $c_{b}$, and drift $c_{d}$.

(1) $${\dot{x}}_{gps}=F_{gps}x_{gps}+w_{gps}.$$

where $x_{gps}={\left[x,\dot{x},\;\ddot{x},\;y,\dot{y},\;\ddot{y},\;z,\dot{z},\;\ddot{z},\;c_{b},\;c_{d}\right]}^{\prime }$ and the state variable $x_{gps}$ denotes the error state,

$$F_{gps}=\left[\begin{array}{c} \begin{array}{ccc} F_{X} & O_{3\times 3} & O_{3\times 3}\\ O_{3\times 3} & F_{Y} & O_{3\times 3}\\ O_{3\times 3} & O_{3\times 3} & F_{Z} \end{array}\;\begin{array}{c} O_{3\times 3}\\ O_{3\times 3}\\ O_{3\times 3} \end{array}\\ \begin{array}{ccc} O_{2\times 2} & O_{2\times 2} & O_{2\times 2} \end{array}\;\;\;\;F_{W\;\;} \end{array}\right],\;\;\;w_{gps}=\left[\begin{array}{c} \begin{array}{c} w_{X}\\ w_{Y}\\ w_{Z} \end{array}\\ w_{W} \end{array}\right],\;F_{X}=F_{Y}=F_{Z}=\left[\begin{array}{ccc} 0 & 1 & 0\\ 0 & 0 & 1\\ 0 & 0 & 0 \end{array}\right],\;F_{W}=\left[\begin{array}{cc} 0 & 1\\ 0 & 0 \end{array}\right],\;\;\;\;w_{X}=\left[\begin{array}{c} 0\\ 0\\ \;w_{x} \end{array}\right],\;w_{Y}=\left[\begin{array}{c} 0\\ 0\\ w_{y} \end{array}\right],w_{Z}=\left[\begin{array}{c} 0\\ 0\\ w_{z} \end{array}\right],\;w_{W}=\left[\begin{array}{c} w_{b}\\ w_{d} \end{array}\right],$$

and $w_{x},\;w_{y},w_{z},\;w_{b},w_{d}$ are independent white noises.

Pseudo range is the distance between the GPS satellite and the receiver. The difference between the measured pseudo range $\rho_{i}$ and the estimated pseudo range $\hat{\rho_{i}}$ is used as the measurement in the Kalman filter for i-th GPS satellite

$$z_{i}=\hat{\rho_{i}}-\rho_{i}=H_{i}x_{gps}+v_{i}$$

where $H_{i}=\left[a_{xi}\;0\;0\;a_{yi}\;0\;0\;a_{zi}\;0\;0\;1\;0\right],\;a_{xi}=\frac{x_{i}-{\hat{x}}_{u}}{{\hat{r}}_{i}}$, $a_{yi}=\frac{y_{i}-{\hat{y}}_{u}}{{\hat{r}}_{i}}$, $a_{zi}=\frac{z_{i}-{\hat{z}}_{u}}{{\hat{r}}_{i}}$, ${\hat{r}}_{i}=\sqrt{{\left(x_{i}-{\hat{x}}_{u}\right)}^{2}+{\left(y_{i}-{\hat{y}}_{u}\right)}^{2}+{\left(z_{i}-{\hat{z}}_{u}\right)}^{2}}$, $\left(x_{i},y_{i},z_{i}\right)$ is the position of i-th GPS satellite, $\left({\hat{x}}_{u},{\hat{y}}_{u},z_{u}\right)$ is the estimated user position, and $v_{i}$ is the white noise. The whole measurement equation is given as follows:

(2) $$z_{gps}=H_{gps}x_{gps}+v_{gps}$$

where $z_{gps}={\left[z_{1}\;z_{2}\;\cdots \;z_{m}\right]}^{\prime }$, $H_{gps}={[{H^{\prime }}_{1}\;{H^{\prime }}_{2}\;\cdots \;{H^{\prime }}_{m}]}^{\prime },v_{gps}={\left[v_{1}\;v_{2}\;\cdots \;v_{m}\right]}^{\prime }$.

From the dynamic Equation (1) and the measurement (2), the accelerations $\ddot{x},\;\ddot{y},\;\mathrm{and}\;\ddot{z}$ can be estimated using the Kalman filter [21].

### 2.3. AHRS

AHRS provides the attitude and heading and thus, the direction cosine matrix $C_{b}^{n}$ can be calculated uniquely if the rotation sequence of roll, pitch, and heading is pre-defined. Many approaches have been proposed for AHRS [22,23,24,25] using accelerometer, gyroscope, and magnetometer. Accelerometers provide roll and pitch, and magnetometers provide heading. Hence the roll, pitch, and heading obtained from accelerometers and magnetometers can be compared with those from the gyroscope, and thus the Kalman filter can be used to estimate attitude and heading.

In many cases, quaternion is used to avoid deadlock and to save time. Quaternion q is defined as one scalar and a three- or four-dimensional vector as follows:

$$q=q_{0}+q_{1}i+q_{2}j+q_{3}k=[q_{0}\;q_{1}\;q_{2}\;q_{3}{]}^{\prime }$$

The direction cosine matrix$\;C_{b}^{n}$ is related with the quaternion q as in (3).

(3) $$C_{b}^{n}=\left[\begin{array}{ccc} q_{0}^{2}+q_{1}^{2}-q_{2}^{2}-q_{3}^{2} & 2\left(q_{1}q_{2}+q_{0}q_{3}\right) & 2\left(q_{1}q_{3}-q_{0}q_{2}\right)\\ 2\left(q_{1}q_{2}-q_{0}q_{3}\right) & q_{0}^{2}-q_{1}^{2}+q_{2}^{2}-q_{3}^{2} & 2\left(q_{2}q_{3}+q_{0}q_{1}\right)\\ 2\left(q_{1}q_{3}+q_{0}q_{2}\right) & 2\left(q_{2}q_{3}-q_{0}q_{1}\right) & q_{0}^{2}-q_{1}^{2}-q_{2}^{2}+q_{3}^{2} \end{array}\right]$$

The quaternion q is updated as the following differential equation

$$\dot{q}=\frac{1}{2}Wq$$

where $W=\left[\begin{array}{cccc} 0 & -\omega_{x} & -\omega_{y} & -\omega_{z}\\ \omega_{x} & 0 & \omega_{z} & -\omega_{y}\\ \omega_{y} & -\omega_{z} & 0 & \omega_{x}\\ \omega_{z} & \omega_{y} & -\omega_{x} & 0 \end{array}\right]$.

The direction cosine matrix $C_{b}^{n},$ which is the output of AHRS, can be described as

$${\hat{C}}_{b}^{n}=\left(I+\Psi \right)C_{b}^{n}$$

where $C_{b}^{n}$ is the true direction cosine matrix, and Ψ denotes the orientation error of AHRS and is a skew symmetric matrix as follows:

(4) $$\Psi =\left[\begin{array}{ccc} 0 & -\delta \psi & \delta \theta \\ \delta \psi & 0 & -\delta \phi \\ -\delta \theta & \delta \phi & 0 \end{array}\right],\;\;\left[\begin{array}{c} \delta \phi \\ \delta \theta \\ \delta \psi \end{array}\right]=\left[\begin{array}{c} \delta \phi \sim N\left(0,\sigma_{\phi }^{2}\right)\\ \delta \theta \sim N\left(0,\sigma_{\theta }^{2}\right)\\ \delta \psi \sim N\left(0,\sigma_{\psi }^{2}\right) \end{array}\right]$$

where ϕ is roll, θ is pitch, and ψ is heading. As discussed in Section 2.1, it is assumed that IMU calibration and initial alignment is performed in advance before AHRS block as in Figure 1. Thus deterministic and some random errors are compensated in the IMU calibration and initial alignment block. Then the remaining orientation error δϕ, δθ, and δψ can be assumed to have Gaussian distribution as in (4).

## 3. Definition of the Decision Variables and the Structure of the Second-half of the Proposed Direct GPS Spoofing Detection

This section describes the acceleration error equation for the direct GPS spoofing detection and defines two decision variables to decide whether a spoofing signal exists or not. One decision variable is the magnitude of the horizontal acceleration error and the other is the magnitude of the north (or east) direction acceleration error. The probability density functions and the thresholds for spoofing detection are given for the two decision variables.

### 3.1. Acceleration Error Equation

The acceleration estimated from the GPS receiver is described as follows,

(5) $${\hat{f}}_{gps}^{n}=f_{gps}^{n}+\epsilon_{gps}^{n}$$

where $f_{gps}^{n}$ is the true moving acceleration (plus spoofing acceleration if any) in the navigation frame and $\epsilon_{gps}^{n}$ is the white noise.

The specific force measured from the accelerometers ${\hat{f}}_{acc}^{b}$ in the body frame is transformed into the navigation frame ${\hat{f}}_{acc}^{n}$ by using the direction cosine matrix ${\hat{C}}_{b}^{n}$ obtained from the AHRS as follows,

(6) $${\hat{f}}_{acc}^{n}={\hat{C}}_{b}^{n}{\hat{f}}_{acc}^{b}=\left(C_{b}^{n}+\Psi C_{b}^{n}\right)\left(f_{acc}^{b}+\epsilon_{acc}^{b}\right)\approx f_{acc}^{n}+\Psi f_{acc}^{n}+\epsilon_{acc}^{n}$$

where $f_{acc}^{n}$ is the true specific force, Ψ is defined in (4), and $\epsilon_{acc}^{n}$ is the white noise of accelerometers. The Coriolis effect is assumed to be negligible with the assumption of low moving velocity for brevity. Notice that the z-component of $f_{acc}^{n}$ contains the gravity.

The acceleration error equation is described from the difference of ${\hat{f}}_{gps}^{n}$ and ${\hat{f}}_{acc}^{n}$ as follows:

(7) $$z={\hat{f}}_{gps}^{n}-{\hat{f}}_{acc}^{n}$$

### 3.2. Decision Variable $z_{mag}$ as the Magnitude of the Horizontal Acceleration Error

Suppose that the hypothesis$\;H_{0}$ denotes the case of the absence of the spoofing signal, and $H_{1}$ denotes the case of the presence of the spoofing signal. For hypothesis$\;H_{0}$, the acceleration error is denoted as$\;z_{0}$, and for hypothesis $H_{1}$, the acceleration error is denoted as $z_{1}.$ Then, $z_{0}$ and $z_{1}$ can be described from Equations (5) and (6) as follows:

(8) $$z_{0}={\hat{f}}_{gps}^{n}-{\hat{f}}_{acc}^{n}=\epsilon_{gps}^{n}-\Psi f_{acc}^{n}-\epsilon_{acc}^{n}=\left[\begin{array}{c} z_{0n}\\ z_{0e}\\ z_{0d} \end{array}\right]$$

(9) $$z_{1}={\hat{f}}_{gps,spoofed}^{n}-{\hat{f}}_{acc}^{n}=f_{s}^{n}+\epsilon_{gps}^{n}-\Psi f_{acc}^{n}-\epsilon_{acc}^{n}\;=\left[\begin{array}{c} f_{s,n}^{n}+\epsilon_{gps,n}^{n}-\delta \psi f_{acc,e}^{n}+\delta \theta f_{acc,d}^{n}-\epsilon_{acc,n}^{n}\\ f_{s,e}^{n}+\epsilon_{gps,e}^{n}+\delta \psi f_{acc,n}^{n}-\delta \phi f_{acc,d}^{n}-\epsilon_{acc,e}^{n}\\ f_{s,d}^{n}+\epsilon_{gps,d}^{n}-\delta \theta f_{acc,n}^{n}+\delta \phi f_{acc,e}^{n}-\epsilon_{acc,d}^{n} \end{array}\right]=\left[\begin{array}{c} z_{1n}\\ z_{1e}\\ z_{1d} \end{array}\right]$$

where $f_{s,n}^{n},\;f_{s,e}^{n},\;f_{s,d}^{n}$ are north, east, down components of spoofing acceleration.

Only the x, y components of the acceleration error equation (8), (9) are used in this study. The acceleration errors $z_{0}$ and $z_{1}$ are expressed in the navigation frame and the superscript *n* will be omitted henceforth, for brevity.

In Equation (9), the random variables $z_{1n}$ and $z_{1e}$, which are north and east components of $z_{1},$ have Gaussian distribution and the probability density function (PDF) is as follows,

(10) $$z_{1n}:\;f_{Z_{1n}}\left(z\right)=\frac{1}{\sqrt{2\pi }\sigma_{n}}e^{-\frac{{\left(z-f_{s,n}\right)}^{2}}{2\sigma_{n}^{2}}}\;\;\mathrm{where}\;\sigma_{n}^{2}=\epsilon_{gps,n}^{2}+\sigma_{\psi }^{2}f_{acc,e}^{2}+\sigma_{\theta }^{2}f_{acc,d}^{2}+\epsilon_{acc,n}^{2}$$

(11) $$z_{1e}:\;f_{Z_{1e}}\left(z\right)=\frac{1}{\sqrt{2\pi }\sigma_{e}}e^{-\frac{{\left(z-f_{s,e}\right)}^{2}}{2\sigma_{e}^{2}}}\;\;\mathrm{where}\;\sigma_{e}^{2}=\epsilon_{gps,e}^{2}+\sigma_{\psi }^{2}f_{acc,n}^{2}+\sigma_{\phi }^{2}f_{acc,d}^{2}+\epsilon_{acc,e}^{2}$$

#### 3.2.1. Probability Density Function of the Magnitude of the Horizontal Acceleration Error $z_{mag}$

$z_{1mag}$ is defined as the magnitude of the horizontal component of $z_{1}$ as follows: 

$z_{1mag}=\sqrt{z_{1n}^{2}+z_{1e}^{2}}$, where $z_{1n}\sim N\left(f_{s,n},\sigma_{n}^{2}\right)\;\mathrm{and}\;z_{1e}\sim N\left(f_{s,e},\sigma_{e}^{2}\right)\;$with $\sigma_{n}^{2}$ and $\sigma_{e}^{2}$ being defined in (10) and (11).

The variances $\sigma_{n}^{2}$ and $\sigma_{e}^{2}$ have different values and depend on the AHRS attitude accuracy times moving acceleration. Thus $\sigma_{n}^{2}$ and $\sigma_{e}^{2}$ are time-varying if the moving acceleration varies with time.

The PDF of the $z_{1mag}$ could not be found in the literature and thus it was derived in this paper and called as Shim PDF. Lemma 1 shows the PDF of$\;z_{1mag}.$

**Lemma** **1.** ***(Shim PDF)** Consider the independent Gaussian random variables*X*and*Y*with*$X\sim N\left(m_{1},\sigma_{1}^{2}\right)\;and\;Y\sim N\left(m_{2},\sigma_{2}^{2}\right)$*. Then, the magnitude*$Z=\sqrt{X^{2}+Y^{2}}$*has the following PDF:*

(12) $$f_{Z}\left(z\right)=\frac{z}{\sigma_{1}\sigma_{2}}\exp\left[-\frac{1}{4}\left(\frac{1}{\sigma_{1}^{2}}+\frac{1}{\sigma_{2}^{2}}\right)\left(z^{2}+\alpha^{2}\right)\right]I_{1}\left(z\right),\;z\geq 0$$

*where*$I_{1}\left(z\right)=\frac{1}{2\pi }\int_{0}^{2\pi }\exp[\frac{1}{4}\left(\frac{1}{\sigma_{2}^{2}}-\frac{1}{\sigma_{1}^{2}}\right)z^{2}\cos\left(2\theta \right)+\sqrt{{\left(\frac{m_{1}}{\sigma_{1}^{2}}\right)}^{2}+{\left(\frac{m_{2}}{\sigma_{2}^{2}}\right)}^{2}}\cdot z\cos\left(\theta -\phi \right)]d\theta$*, and*$\alpha^{2}=\frac{2\left(\sigma_{2}^{2}m_{1}^{2}+\sigma_{1}^{2}m_{2}^{2}\right)}{\sigma_{1}^{2}+\sigma_{2}^{2}}=\frac{2\left(\frac{m_{1}^{2}}{\sigma_{1}^{2}}+\frac{m_{2}^{2}}{\sigma_{2}^{2}}\right)}{\frac{1}{\sigma_{1}^{2}}+\frac{1}{\sigma_{2}^{2}}}$, $\phi ={tan}^{-1}\left(\frac{m_{2}\sigma_{1}^{2}}{m_{1}\sigma_{2}^{2}}\right)={tan}^{-1}\left(\frac{\frac{m_{2}}{\sigma_{2}^{2}}}{\frac{m_{1}}{\sigma_{1}^{2}}}\right).$

**Proof.**  The variable change for the independent Gaussian random variables *X* and *Y* is as

$$U=\frac{X}{s},\;V=\frac{Y}{s}\;\mathrm{where}\;U∼N\left(\frac{m_{1}}{s},\frac{\sigma_{1}^{2}}{s^{2}}\right),\;V∼N\left(\frac{m_{2}}{s},\frac{\sigma_{2}^{2}}{s^{2}}\right),\;\mathrm{and}\;s=\sqrt{\sigma_{1}^{2}+\sigma_{2}^{2}\;}$$

Defining $\mu_{1}=\frac{m_{1}}{s}\;\mathrm{and}\;\mu_{2}=\frac{m_{2}}{s},\;b=\frac{\sigma_{1}^{2}-\sigma_{2}^{2}}{\sigma_{1}^{2}+\sigma_{2}^{2}}$, it can be understood that $U∼N\left(\mu_{1},\frac{1+b}{2}\right),\;V∼N\left(\mu_{2},\frac{1-b}{2}\right)\;\mathrm{and}-1\leq b\leq 1.$

The idea of using *s* and *b* above comes from Hoyt’s paper [26]. Consider the joint PDF $f_{UV}\left(u,v\right).$

$$\begin{array}{ll} f_{UV}\left(u,v\right)=f_{U}\left(u\right)f_{V}\left(v\right) & =\frac{1}{\sqrt{\pi \left(1+b\right)}}\exp\left[-\frac{{\left(u-\mu_{1}\right)}^{2}}{1+b}\right]\cdot \frac{1}{\sqrt{\pi \left(1-b\right)}}\exp\left[-\frac{{\left(v-\mu_{2}\right)}^{2}}{1-b}\right]\\ & =\frac{1}{\pi \sqrt{1-b^{2}}}\exp\left[-\frac{{\left(u-\mu_{1}\right)}^{2}}{1+b}-\frac{{\left(u-\mu_{2}\right)}^{2}}{1-b}\right] \end{array}$$

Defining $R=\sqrt{U^{2}+V^{2}}$, the PDF $f_{R}\left(r\right)$ can be obtained as follows:

$$\begin{array}{ll} f_{R}\left(r\right) & =\int_{0}^{2\pi }f_{UV}\left(u,v\right)rd\theta \\ & =\int_{0}^{2\pi }f_{UV}\left(r\cos\theta ,r\sin\theta \right)rd\theta \\ & =\int_{0}^{2\pi }\frac{1}{\pi \sqrt{1-b^{2}}}\exp\left[-\frac{{\left(r\cos\theta -\mu_{1}\right)}^{2}}{1+b}-\frac{{\left(r\sin\theta -\mu_{2}\right)}^{2}}{1-b}\right]rd\theta \end{array}$$

By algebraic manipulation of the above equation, $f_{R}\left(r\right)=\frac{r}{\pi \sqrt{1-b^{2}}}\;\exp\left[-\frac{r^{2}+\beta^{2}}{1-b^{2}}\right]\int_{0}^{2\pi }\exp\left[\frac{br^{2}\cos\left(2\theta \right)+rA\cos\left(\theta -\phi \right)}{1-b^{2}}\right]d\theta$ where $\beta^{2}=\left(1-b\right)\mu_{1}^{2}+\left(1+b\right)\mu_{2}^{2},\;\;A=2\sqrt{{\left(1-b\right)}^{2}\mu_{1}^{2}+{\left(1+b\right)}^{2}\mu_{2}^{2}},\;\phi =\tan^{-1}\left(\frac{\left(1+b\right)\mu_{2}}{\left(1-b\right)\mu_{1}}\right).$

From the relation between random variables Z and R as Z=sR, the PDF of Z can be obtained from $f_{Z}\left(z\right)=\frac{1}{s}f_{R}\left(\frac{z}{s}\right)$ as:

(13) $$f_{Z}\left(z\right)=\frac{1}{s}\cdot \frac{z/s}{\pi \sqrt{1-b^{2}}}\;\exp\left[-\frac{{\left(\frac{z}{s}\right)}^{2}+\beta^{2}}{1-b^{2}}\right]\int_{0}^{2\pi }\exp\left[\frac{b{\left(\frac{z}{s}\right)}^{2}\cos\left(2\theta \right)+\left(\frac{z}{s}\right)A\cos\left(\theta -\phi \right)}{1-b^{2}}\right]d\theta$$

From algebraic manipulation of Equation (13), $\frac{1}{s}\cdot \frac{\frac{z}{s}}{\pi \sqrt{1-b^{2}}}\;$=$\;\frac{z}{\sigma_{1}\sigma_{2}}\cdot \frac{1}{2\pi }$ and $\exp\left[-\frac{{\left(\frac{z}{s}\right)}^{2}+\beta^{2}}{1-b^{2}}\right]=\exp\left[-\frac{1}{4}\left(\frac{1}{\sigma_{1}^{2}}+\frac{1}{\sigma_{2}^{2}}\right)\left(z^{2}+\alpha^{2}\right)\right]$ is obtained, and the integral term in Equation (13) becomes the integral term of $I_{1}\left(z\right),\;$which results in Equation (12). □

Rayleigh PDF and Rice PDF are well-known PDFs as the relation to Gaussian, and those two PDFs are special cases of Equation (12), which becomes Rayleigh PDF with the condition of $m_{1}=m_{2}=0$ and $\sigma_{1}=\sigma_{2}$, and becomes Rice PDF with the condition of $\sigma_{1}=\sigma_{2}$.

Defining $z_{0mag}$ as the magnitude of the horizontal component of $z_{0}$ as follows, $z_{0mag}=\sqrt{z_{0n}^{2}+z_{0e}^{2}}$, where $z_{1n}\sim N\left(0,\sigma_{n}^{2}\right),\;\;z_{1e}\sim N\left(0,\sigma_{e}^{2}\right).\;$

The PDF of$\;z_{0mag}$ can be obtained from Lemma 1 with $m_{1}=m_{2}=0$ and the result is shown in Corollary 2.

**Corollary** **2.** *Consider the independent Gaussian random variables* X *and* Y *with* $X\sim N\left(0,\sigma_{1}^{2}\right)\;\mathrm{and}\;Y\sim N\left(0,\sigma_{2}^{2}\right)$ *. Then the magnitude* $Z=\sqrt{X^{2}+Y^{2}}$ *has the following PDF:*

(14) $$f_{Z}\left(z\right)=\frac{z}{\sigma_{1}\sigma_{2}}\exp\left[-\frac{1}{4}\left(\frac{1}{\sigma_{1}^{2}}+\frac{1}{\sigma_{2}^{2}}\right)z^{2}\right]I_{0}\left(\frac{1}{4}\left(\frac{1}{\sigma_{2}^{2}}-\frac{1}{\sigma_{1}^{2}}\right)z^{2}\right),\;z\geq 0$$

*where* $I_{0}\left(z\right)=\frac{1}{\pi }\int_{0}^{\pi }e^{z\cos\theta }d\theta$ *,*

**Proof.**  Equation (14) can be obtained easily from the PDF in Equation (12) with $m_{1}=m_{2}=0$ and manipulation in the $I_{0}\left(z\right)$ part. □

Equation (14) can be found in Reference [27] without proof.

#### 3.2.2. Threshold to Detect GPS Spoofing for the Decision Variable $z_{mag}$

In this study, the probability of false alarm is used to obtain the threshold for the detection of spoofing. The threshold $\gamma_{mag}$ to detect GPS spoofing is defined according to the pre-defined probability of false alarm $P_{fa}\;\mathrm{as}\;\mathrm{follows}:$

(15) $$\mathrm{prob}\left\{z_{0mag}\geq \gamma_{mag}\right\}=\mathrm{prob}\left\{z_{mag}\geq \gamma_{mag}|H_{0}\right\}=P_{fa}$$

where

(16) $$z_{mag}=\sqrt{z_{n}^{2}+z_{e}^{2}}$$

and the probability is calculated from the integral of equation (14) from $\gamma_{mag}$ to infinity.

Whether a spoofing signal exists or not is decided by the following decision rule:

$$z_{mag}\begin{array}{c} H_{1}\\ ≷\\ H_{0} \end{array}\;\gamma_{mag}$$

The variable $z_{mag}$ above is said to be a decision variable since it is used to decide whether there is a spoofing signal or not.

### 3.3. Decision Variable $z_{absN}$ (or $z_{absE}$) as the Magnitude of the North (or East) Direction Acceleration Error

#### 3.3.1. Probability Density Function of the Magnitude of The North (or East) Acceleration Error $z_{absN}$ (or $z_{absN}$)

Defining $z_{1absN}$ and $z_{0absN}$ as the magnitude of the north component of $z_{1}$ and $z_{0}$, respectively, $z_{1absN}=\left|z_{1n}\right|$ and $z_{0absN}=\left|z_{0n}\right|$ (similarly, $z_{1absE}=\left|z_{1e}\right|\;\mathrm{and}\;z_{0absE}=\left|z_{0e}\right|).$

The PDF of $z_{1absN}$ (or $z_{1absE})\;$and $z_{0absN}$ (or $z_{0absE})$ can be obtained as Equations (17) and (18), which are called folded Gaussian [28], since $z_{1n}$ and $z_{0n}\;$have Gaussian density functions as $z_{1n}\sim N\left(f_{s,n},\sigma_{n}^{2}\right)$ and $z_{0n}\sim N\left(0,\sigma_{n}^{2}\right)$.

(17) $$f_{Z_{1absN}}\left(z\right)=\frac{1}{\sqrt{2\pi }\sigma_{n}}e^{-\frac{{\left(z-f_{s,n}\right)}^{2}}{2\sigma_{n}^{2}}}+\frac{1}{\sqrt{2\pi }\sigma_{n}}e^{-\frac{{\left(z+f_{s,n}\right)}^{2}}{2\sigma_{n}^{2}}},\;z\geq 0$$

(18) $$f_{Z_{0absN}}\left(z\right)=\frac{2}{\sqrt{2\pi }\sigma_{n}}e^{-\frac{z^{2}}{2\sigma_{n}^{2}}},\;z\geq 0$$

#### 3.3.2. Threshold to Detect GPS Spoofing for the Decision Variable $z_{absN}$

The threshold $\gamma_{absN}$ to detect GPS spoofing is defined according to the pre-defined probability of false alarm $P_{fa}\;\mathrm{as}\;\mathrm{follows}:$

(19) $$\mathrm{prob}\left\{z_{0absN}\geq \gamma_{absN}\right\}=\mathrm{prob}\left\{z_{absN}\geq \gamma_{absN}|H_{0}\right\}=P_{fa}$$

here

(20) $$z_{absN}=\left|z_{n}\right|$$

and the probability is calculated from the integral of Equation (18) from $\gamma_{absN}$ to infinity. Whether a spoofing signal exists or not is decided by the following decision rule:

$$z_{absN}\begin{array}{c} H_{1}\\ ≷\\ H_{0} \end{array}\;\gamma_{absN}$$

### 3.4. The Structure of the Second-Half of the Proposed Direct GPS Spoofing Detection

**Figure 2:**
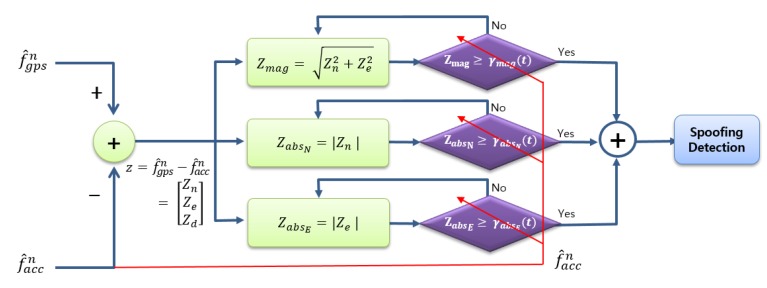
The Structure of the Second-half of the proposed direct GPS spoofing detection after Figure 1.

This subsection shows the structure of the proposed second-half of direct GPS spoofing detection method in Figure 2, which is drawn after the rightmost signal in Figure 1. The analysis of the proposed structure shown in Figure 2 will be given in Section 4 and Section 5 in detail.

In Section 4, it will be observed that the decision variable $z_{absN}$ (or $z_{absE}$) shows a higher detection probability than $z_{mag}$ in the condition that both moving acceleration and spoofing acceleration head within roughly 25° from the north (or east). Section 5 shows that when DMSA is used for performance measure, the decision variable $z_{absN}$ (or $z_{absE}$) shows a smaller DMSA than $z_{mag}$ in the condition that both moving acceleration and spoofing acceleration head within roughly 25° from the north–south direction (or east–west direction). From these results, a direct GPS spoofing detection method is proposed as follows: 

If any of the three decision variables $z_{mag}$, $z_{absN,}$ and $z_{absE}$ are larger than or equal to the corresponding thresholds, then a spoofing signal is declared to exist.

Note that the threshold $\gamma_{mag}\left(t\right),\;\gamma_{absN}\left(t\right),\;\mathrm{and}\;\gamma_{absE}\left(t\right)$ in Figure 2 are time-varying, not constant. The threshold $\gamma_{mag}\left(t\right)$ is obtained from Equation (15) given the probability of false alarm $P_{fa}$, where the PDF is Equation (14) with $\sigma_{1}=\sigma_{n}$ and $\sigma_{2}=\sigma_{e}$. The north and east variances $\sigma_{n}^{2}$ and $\sigma_{e}^{2}$ given in Equations (10) and (11) contain the moving acceleration and thus $\sigma_{n}^{2}$ and $\sigma_{e}^{2}$ are time-varying, which is why $\gamma_{mag}\left(t\right)$ is time-varying. The threshold $\gamma_{absN}\left(t\right)$ is obtained from Equation (18) and the PDF contains $\sigma_{n}$, which is time-varying. Thus $\gamma_{absN}\left(t\right)$ depends on the moving acceleration and becomes time-varying. Similarly $\gamma_{absE}\left(t\right)$ is time-varying. The red line and arrow in Figure 2 means that the threshold $\gamma_{mag}\left(t\right),\;\gamma_{absN}\left(t\right),\;\mathrm{and}\;\gamma_{absE}\left(t\right)$ depend on the moving acceleration ${\hat{f}}_{acc}^{n}.$

## 4. Performance Analysis of the Decision Variables using the Probability of Detection

This section shows the performance of the proposed direct GPS spoofing detection method for the two decision variables $z_{mag}$ and $z_{absN}$ (or $z_{absE}$) which are defined in Section 3.

### 4.1. Detection Threshold According to Moving Acceleration

Suppose that the probability of false alarm $P_{fa}$ is pre-defined. Then, spoofing detection thresholds $\gamma_{mag}$ and $\gamma_{absN}$(or $\gamma_{absE}$) are determined according to $P_{fa}$ as in Equations (15) and (19). Taking the PDF Equations (14) and (18) into account, Equations (21) and (22) are obtained from Equations (15) and (19) to further obtain $\gamma_{mag}$ and $\gamma_{absN}$.

(21) $$\mathrm{prob}\left\{z_{mag}\geq \gamma_{mag}|H_{0}\right\}=\int_{\gamma_{mag}}^{\infty }\frac{z}{\sigma_{n}\sigma_{e}}\exp\left[-\frac{1}{4}\left(\frac{1}{\sigma_{n}^{2}}+\frac{1}{\sigma_{e}^{2}}\right)z^{2}\right]I_{0}\left(\frac{1}{4}\left(\frac{1}{\sigma_{e}^{2}}-\frac{1}{\sigma_{n}^{2}}\right)z^{2}\right)dz\;=P_{fa}$$

(22) $$\mathrm{prob}\left\{z_{ansN}\geq \gamma_{absN}|H_{0}\right\}=\int_{\gamma_{absN}}^{\infty }\frac{2}{\sqrt{2\pi }\sigma_{n}}e^{-\frac{z^{2}}{2\sigma_{n}^{2}}}\;dz\;=P_{fa}$$

where $\sigma_{n}^{2}=\epsilon_{gps,n}^{2}+\sigma_{\psi }^{2}f_{acc,e}^{2}+\sigma_{\theta }^{2}f_{acc,d}^{2}+\epsilon_{acc,n}^{2}$ and $\sigma_{e}^{2}=\epsilon_{gps,e}^{2}+\sigma_{\psi }^{2}f_{acc,n}^{2}+\sigma_{\phi }^{2}f_{acc,d}^{2}+\epsilon_{acc,e}^{2}$.

To see the detection performance result clearly, the vertical moving acceleration is supposed to be zero and the gravity is compensated before the acceleration error is obtained. Thus the following variances in Equation (23) are used in the simulation from now on.

(23) $$\sigma_{n}^{2}=\epsilon_{gps,n}^{2}+\sigma_{\psi }^{2}f_{acc,e}^{2}+\epsilon_{acc,n}^{2}\;\mathrm{and}\;\;\sigma_{e}^{2}=\epsilon_{gps,e}^{2}+\sigma_{\psi }^{2}f_{acc,n}^{2}+\epsilon_{acc,e}^{2}$$

**Figure 3:**
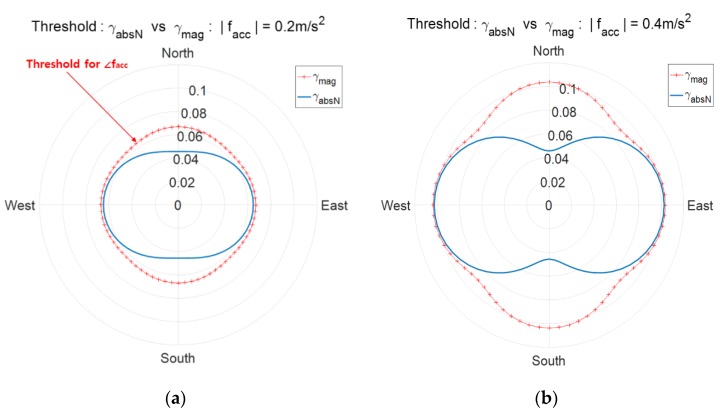
Spoofing detection thresholds $\gamma_{mag}$ and $\gamma_{absN}$ according to the moving acceleration. The distance from the origin is the threshold for the corresponding direction of moving acceleration; (**a**)$\;\left|f_{acc}\right|=0.2\;m/s^{2}$, (**b**) $\left|f_{acc}\right|=0.4\;m/s^{2}$.

The detection thresholds $\gamma_{mag}$ and $\gamma_{absN}$ depend on the variances $\sigma_{n}^{2}$ and $\sigma_{e}^{2}$ which are functions of moving acceleration $f_{acc}$ as in (23). Thus, the detection threshold $\gamma_{mag}$ and $\gamma_{absN}$ are not constant but vary according to the magnitude and direction of the moving acceleration $f_{acc}$. Figure 3 shows detection thresholds $\gamma_{mag}$ and $\gamma_{absN}$ according to the direction of $f_{acc}$ with two cases of magnitude, (a) $\left|f_{acc}\right|=0.2\;m/s^{2}$ and (b) $\left|f_{acc}\right|=0.4\;m/s^{2}$. The threshold is the distance from the origin for the corresponding direction of $f_{acc}$. It is observed that for the same magnitude of moving acceleration, $\gamma_{mag}$ has maximum values in the north and east directions and $\gamma_{absN}$ has the minimum value in the north direction. Similarly, $\gamma_{absN}$ has the minimum value in the east direction.

### 4.2. Effects of Moving Acceleration on the Performance of Spoofing Detection

This subsection analyzes the effects of moving acceleration on the performance of spoofing detection. The effects of moving acceleration, magnitude and direction are separately examined, for both decision variables $z_{mag}$ and $z_{absN}$ which are defined in Section 3.

The probability of detection $P_{d}$ is used for the performance of spoofing detection with the pre-defined probability of false alarm $P_{fa}$. When the detection threshold $\gamma_{mag}$ and $\gamma_{absN}$ are obtained from$\;P_{fa}$, the corresponding detection probabilities $P_{d,mag}$ and $P_{d,absN}$ are defined as follows:

(24) $$P_{d,mag}=\mathrm{prob}\left\{z_{mag}\geq \gamma_{mag}|H_{1}\right\}\;\mathrm{and}\;P_{d,absN}=\mathrm{prob}\left\{z_{absN}\geq \gamma_{absN}|H_{1}\right\}$$

where the probability density functions (12) and (17) are integrated from the detection threshold to infinity.

#### 4.2.1. Performance of Spoofing Detection According to the Magnitude of Moving Acceleration

The probability of detection $P_{d}$ depends on both moving acceleration $f_{acc}\;$and spoofing acceleration $f_{s}$. The effect of the magnitude of moving acceleration is focused on in this subsection.

**Figure 4:**
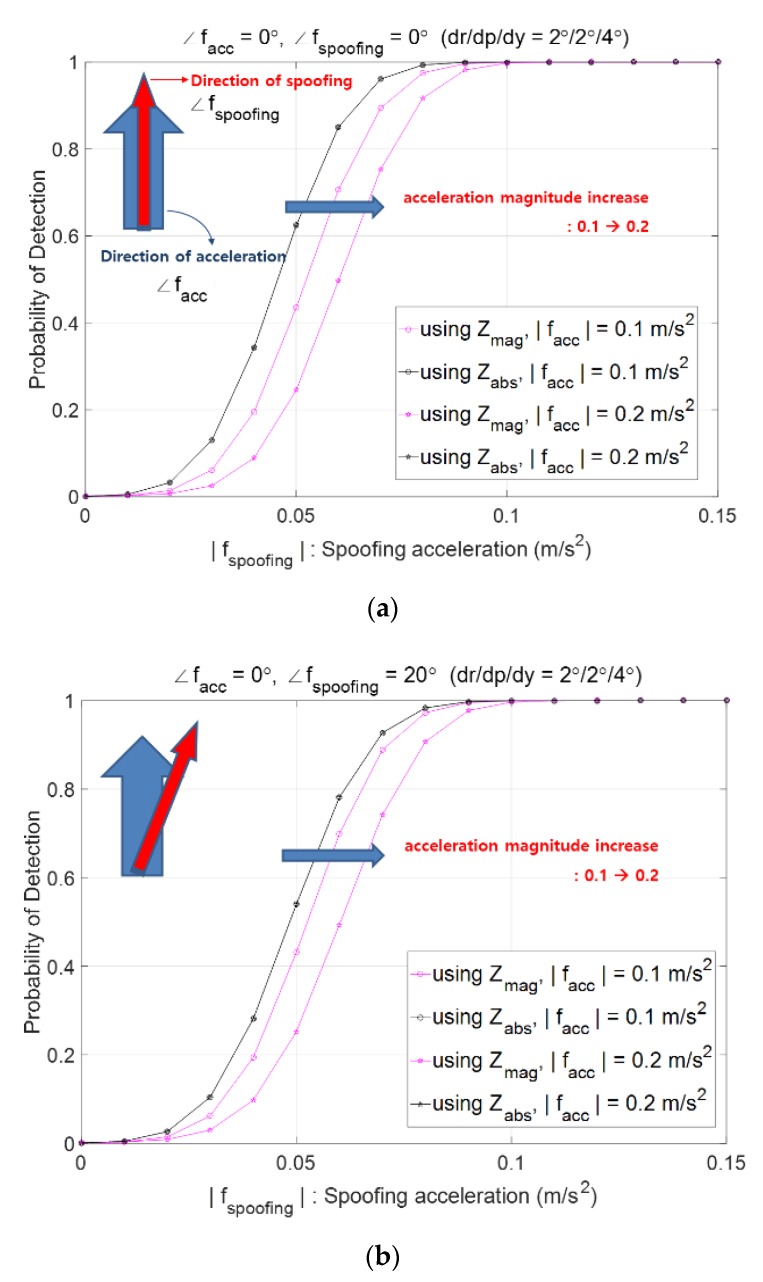
Probabilities of spoofing detection$,\;P_{d,mag}$ and $P_{d,absN}$ when (**a**) Both $f_{acc}\;$ and $f_{s}$ are heading north, (**b**) $f_{acc}\;$ is heading north and $f_{s}\;\mathrm{heading}\;\mathrm{northeast}\;$ 20° (arrow in cyan: moving acceleration $f_{acc}$, narrow arrow in red: spoofing acceleration $f_{s}$ ).

Suppose that the probability of the false alarm is pre-defined as $P_{fa}=0.001$, the AHRS attitude accuracies of roll, pitch, and heading are 2°, 2°, and 4°, respectively, and moving acceleration is heading north. Figure 4 plots $P_{d}$ according to the magnitude of $f_{s}$ and shows that $P_{d}$ increases as $\left|f_{s}\right|$ increases. The big arrow in cyan color in the upper left corner of Figure 4 denotes the moving acceleration $f_{acc}$ and the narrow arrow in red color denotes the spoofing acceleration $f_{s}$. Figure 4a shows the case of both $f_{acc}$ and $f_{s}$ heading north, and plots four curves, two pink in color and two black in color, where the two pink curves show $P_{d,mag}$ and the two black curves show $P_{d,absN}$. The two black curves are same as one, implying that two cases of $\left|f_{acc}\right|=0.1\;m/s^{2}$ and $\left|f_{acc}\right|=0.2\;m/s^{2}$ do not cause any effect on $P_{d,absN},$ while the two pink curves show different results. As $\left|f_{acc}\right|$ changes from $0.1\;m/s^{2}$ to $0.2\;m/s^{2}$, the performance of using $z_{mag}$, which is $P_{d,mag}$, deteriorates. Figure 4a shows that the performance of using $z_{absN}$ is always better than that of using $z_{mag}$ when both $f_{acc}$ and $f_{s}$ head north. Figure 4b shows similar results as Figure 4a when $f_{s}$ heads 20° east from north.

The reason the two black curves are the same in Figure 4 despite two different $\left|f_{acc}\right|$s is because the variance $\sigma_{n}^{2}$ in (10) does not contain $f_{acc,n}$, but $f_{acc,e}$. When $f_{acc}$ heads north, $f_{acc,e}=0$ and thus for a different north speed, the threshold $\gamma_{absN}$ is same, which provides the same $P_{d,absN}.$

#### 4.2.2. Performance of Spoofing Detection According to the Direction of Moving Acceleration

**Figure 5:**
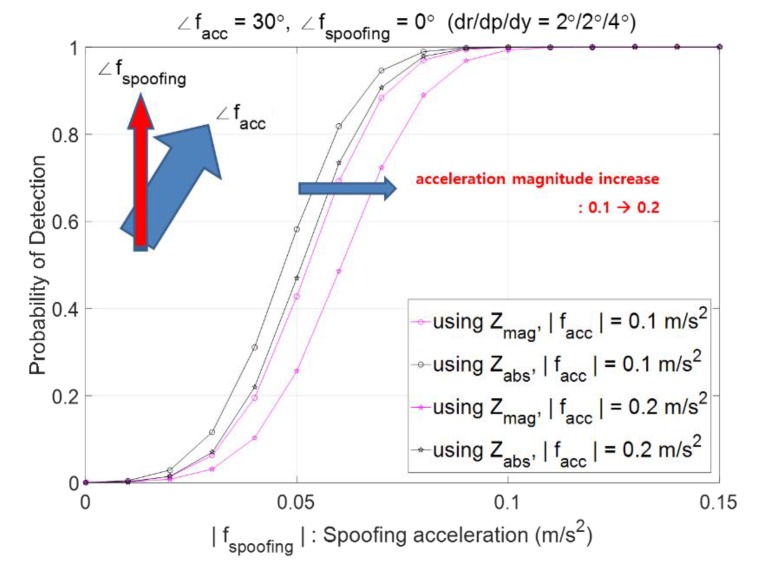
Probability of spoofing detection$,\;P_{d,mag}$ and $P_{d,absN}$ when $f_{acc}$ heads northeast.

In this subsection, the effect of the direction of moving acceleration on the spoofing detection performance is presented. When the direction of $f_{acc}$ changes from north to northeast of 30°, Figure 4a changes into Figure 5, where two black curves are distinct. Since the direction of $f_{acc}$ changes from north to northeast of 30°, the east component $f_{acc,e}$ exists and thus $\sigma_{n}^{2}$ is different for different speeds, which results in a different threshold $\gamma_{absN}\;$and thus different $P_{d,absN}.$ For both $z_{mag}$ and $z_{absN}$ the performance $P_{d}$ deteriorates as $\left|f_{acc}\right|$ increases from $0.1\;m/s^{2}$ to $0.2\;m/s^{2}$. Looking into the black curve of $\left|f_{acc}\right|=0.1\;m/s^{2}$ carefully in Figure 4a and Figure 5, it is observed that $P_{d,absN}$ with $f_{acc}$ heading north is bigger than $P_{d,absN}$ with $f_{acc}$ heading northeast of 30°. For both Figure 4 and Figure 5, the performance of $P_{d,absN}$ is better than that of $P_{d,mag}.$

### 4.3. Effects of Spoofing Acceleration on the Performance of Spoofing Detection

This subsection analyzes the effects of spoofing acceleration on the performance of spoofing detection. The effects of the direction of the spoofing acceleration are examined for both decision variables $z_{mag}$ and $z_{absN}$.

**Figure 6:**
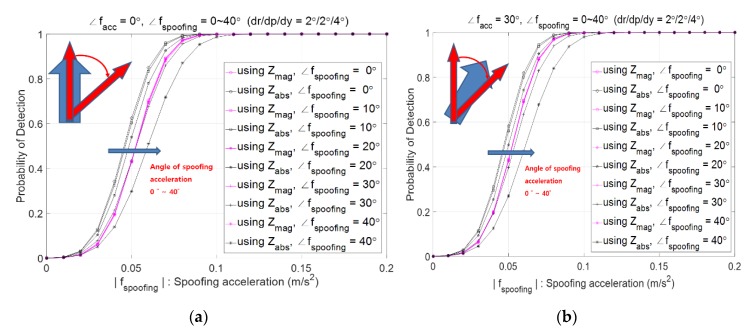
Probability of spoofing detection$,\;P_{d,mag}$ and $P_{d,absN}$ according to ∠$f_{s}$ with $\left|f_{acc}\right|=0.6\;m/s^{2}$, where $f_{s}$ is red color and $f_{acc}$ is cyan color; (**a**) $f_{acc}\;$ is heading north, (**b**) $f_{acc}\;$ is heading 30° east from north.

Figure 6 shows the spoofing detection probability when spoofing direction changes from 0° to 40° from north in the case of $f_{acc}$ heading north as (a) and heading 30° east from north as (b). The horizontal axis is the magnitude of spoofing acceleration. It shows that $P_{d,mag}$ does not depend on the direction of spoofing acceleration while $P_{d,absN}$ decreases as the spoofing direction changes from 0° to 40° from north. This is because the north component of spoofing acceleration decreases as the spoofing direction gets far away from the north. It is observed that $P_{d,absN}$ is greater than $P_{d,mag}$ when the direction of $f_{s}$ is less than 20° for both Figure 6a,b.

### 4.4. Effects of Sensor Accuracy on the Performance of Spoofing Detection

**Figure 7:**
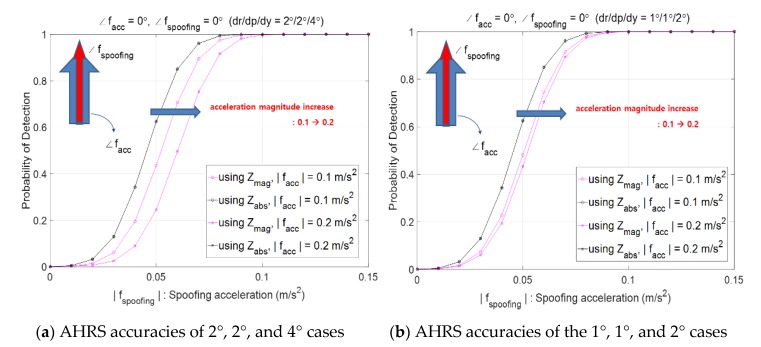
Probability of spoofing detection$,\;P_{d,mag}$ and $P_{d,absN}$ according to Attitude and Heading Reference System (AHRS) accuracy.

**Figure 8:**
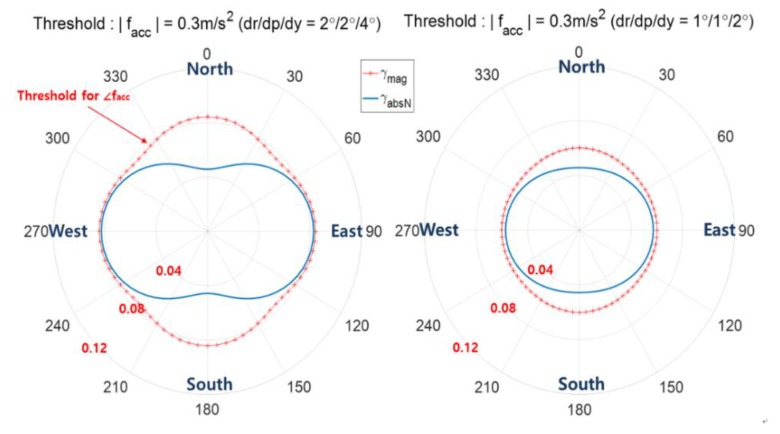
Comparison of spoofing detection threshold according to AHRS accuracy - the angles denote the direction of$\;f_{acc}$, i.e., ∠$f_{acc}$.

This subsection analyzes the effects of sensor accuracy on the performance of spoofing detection. Figure 7a considers AHRS accuracies of 2°, 2°, and 4° for the roll, pitch, and heading errors while Figure 7b considers AHRS accuracies of 1°, 1°, and 2°. It is observed that $P_{d,mag}$(pink color) decreases as the magnitude of $f_{acc}$ increases or AHRS accuracy deteriorates. When both $f_{acc}$ and $f_{s}$ head north, $P_{d,absN}$ is greater than $P_{d,mag}$ in Figure 7. Better the AHRS accuracy, the better is the spoofing detection performance and this can be explained in Figure 8 which shows that with better AHRS accuracy, the threshold is smaller, which results in higher detection performance.

## 5. Performance Analysis of the Decision Variables Using the Detectable Minimum Spoofing Acceleration (DMSA)

This section compares the performance of the proposed direct GPS spoofing detection method for the two decision variables $z_{mag}$ and $z_{absN}$ (or $z_{absE}$) using the minimum threshold of spoofing detection with pre-defined false alarm probability and detection probability.

### 5.1. Spoofing Detection Threshold According to Moving Acceleration

**Figure 9:**
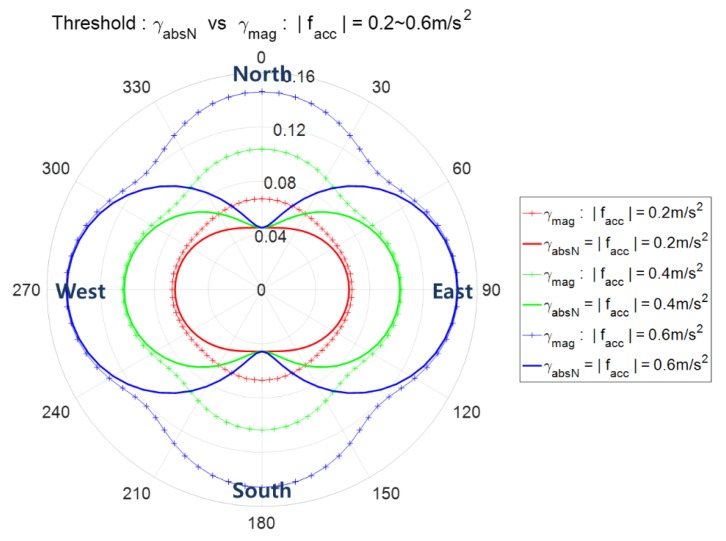
The threshold of spoofing detection $\gamma_{mag}$ and $\gamma_{absN}$ according to $f_{acc},$ the angle denotes the direction of$\;f_{acc}$, i.e., ∠$f_{acc}$.

The detection threshold $\gamma_{mag}$ and $\gamma_{absN}$ according to the direction of $f_{acc}$ for the two cases of $\left|f_{acc}\right|=0.2\;m/s^{2}$ and $\left|f_{acc}\right|=0.4\;m/s^{2}$ are shown in Figure 3, where two decision variables $z_{mag}$ and $z_{absN}$ are used. Figure 9 shows Figure 3a,b together again upon adding the case of $\left|f_{acc}\right|=0.6\;m/s^{2}$ under the condition of $P_{fa}$= 0.001 and the AHRS attitude accuracy of 2/2/4°. Figure 9 shows the exact threshold $\gamma_{mag}$ and $\gamma_{absN}$ and is obtained using Equations (21) and (22) given the pre-defined $p_{fa}$. 

### 5.2. Definition of DMSA

This section defines the detectable minimum spoofing acceleration (DMSA) and compares the DMSA for the decision variables $z_{mag}$ and $z_{absN}$.

**Figure 10:**
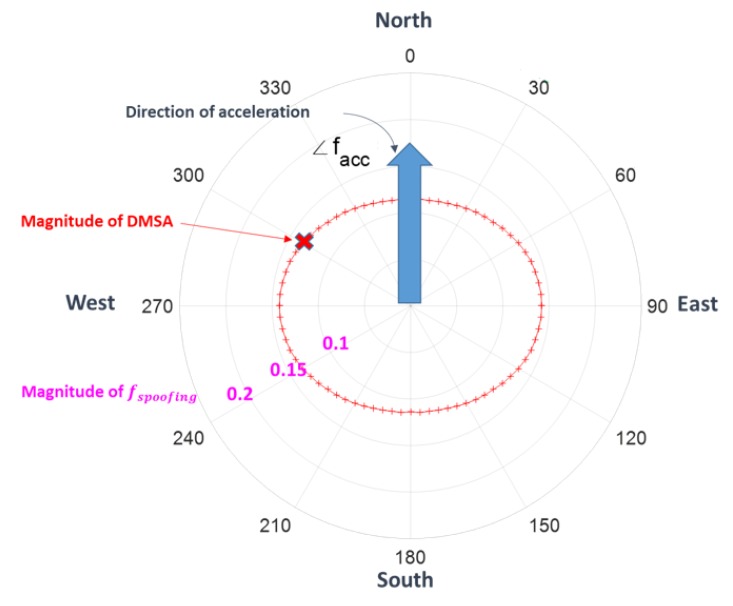
Detectable minimum spoofing acceleration (DMSA), the angle denotes the direction of $f_{s}$, i.e., ∠$f_{s}$.

DMSA is the magnitude of the minimum spoofing acceleration to obtain a pre-defined detection probability $P_{d}\;$given a pre-defined false alarm probability $P_{fa}$. $P_{fa}=0.001$, $P_{d}=0.99$, and AHRS attitude accuracy of 2/2/4° are used for DMSA in the simulations. Figure 10 shows an example of the computation results of DMSA, where the big arrow in cyan color is the moving acceleration $f_{acc}\;$and the angles of 0 through 360° denote the angle of spoofing acceleration $f_{s}$. The contour of red ‘+’ is the set of DMSA for all directions of $f_{s}$. For example, the ‘x’ point means that when $f_{s}$ comes from 330° direction, the DMSA is the distance from the origin to ‘x’ point, which guarantees $P_{d}=0.99.$

#### 5.2.1. Contour of DMSA Using The Decision Variable $z_{mag}$ Depending on The Moving Acceleration 

**Figure 11:**
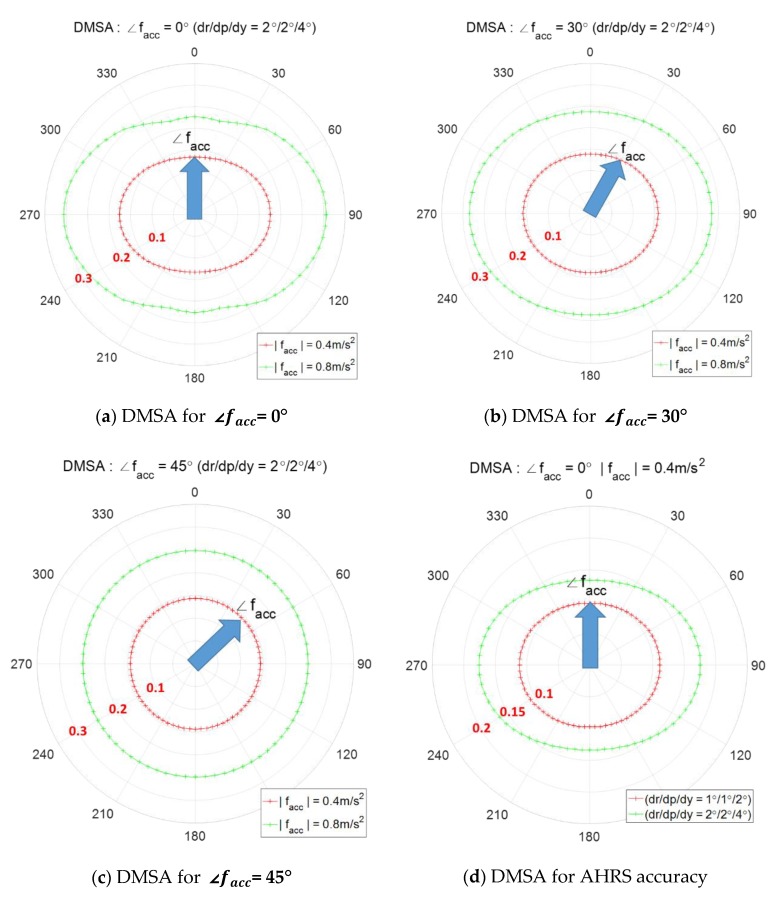
Contour of DMSA using the decision variable$\;z_{mag}$, the angle denotes the direction of$\;f_{s}$, i.e., ∠$f_{s}$; (**a**) ∠$f_{acc}$ = 0°, (**b**) ∠$f_{acc}$ = 30°, (**c**) ∠ $f_{acc}$ = 45°, and (**d**) AHRS accuracy.

For a given DMSA in Figure 10, the values of $P_{fa}$, $P_{d}$, and AHRS attitude accuracy are fixed in advance, and thus the moving acceleration is the only remaining parameter that can affect the DMSA. Figure 11a–c show the contour of DMSA according to ∠$f_{acc}$ of 0°, 30°, and 45°. Greater the |$f_{acc}|$, the bigger the DMSA. The contour of DMSA in Figure 11c appears like a circle since the north and east component of $f_{acc}$ are same and thus$\;\sigma_{n}=\sigma_{e}$. To check the effect of the AHRS accuracy, the DMSA is calculated for two sets of AHRS attitude accuracy of 1/1/2° and 2/2/4° in Figure 11d which shows that the better the accuracy, the smaller the contour of DMSA.

#### 5.2.2. Contour of DMSA Using The Decision Variable $Z_{absN}$ Depending on the Moving Acceleration

**Figure 12:**
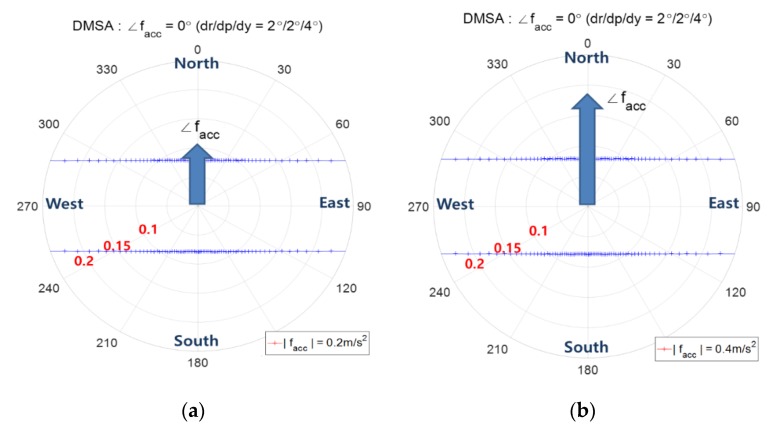
Contour of DMSA using the decision variable $z_{absN}$ (∠$f_{acc}=0{}^{\circ}$ ), the angle denotes the direction of$\;f_{s}$, i.e., ∠$f_{s}$ **;** (**a**) $\left|f_{acc}\right|=0.2\;m/s^{2}$ (**b**) $\left|f_{acc}\right|=0.4\;m/s^{2}$.

**Figure 13:**
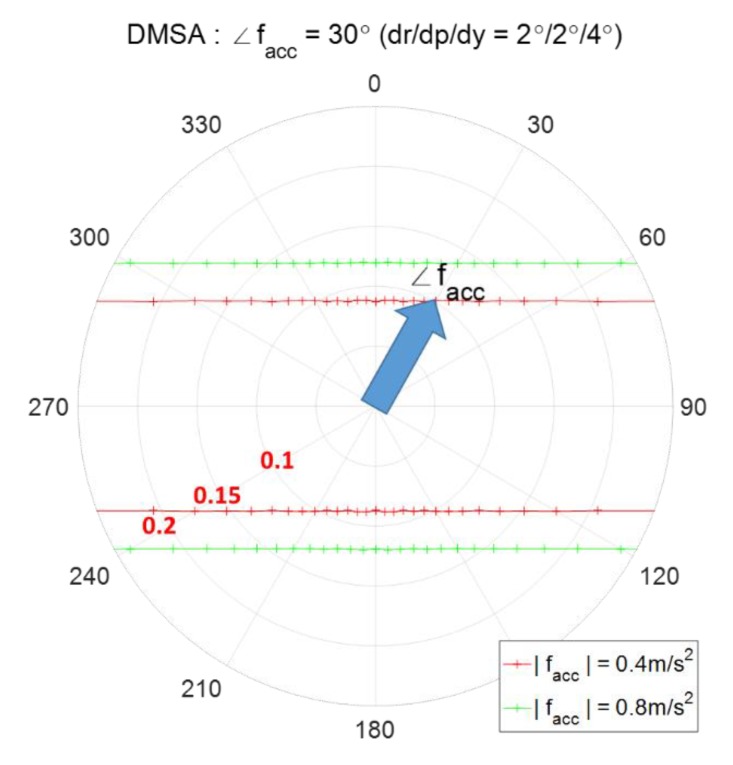
Contour of DMSA using the decision variable $Z_{absN}$ (∠$f_{acc}=30{}^{\circ}$ ), the angle denotes the direction of $f_{s}$, i.e., ∠$f_{s}$.

This subsection shows the contour of DMSA for the decision variable $Z_{absN}=\left|z_{n}\right|$. Figure 12 shows the contour of DMSA for ∠$f_{acc}=0{}^{\circ}.$ When $f_{acc}$ heads north, the variance $\sigma_{n}$ does not depend on |$f_{acc}|$ as shown in Figure 9, so the threshold and DMSA are the same for different magnitudes of $f_{acc}$ as in Figure 12a,b. The contour of DMSA is a line passing through the minimum point of the north direction. Figure 13 shows the contour of DMSA for ∠$f_{acc}=30{}^{\circ}.$ In this case, the east component of $f_{acc}\;$has an effect on the $\sigma_{n}$, which results in a different threshold and DMSA according to different |$f_{acc}|$ as in Figure 13. The bigger the acceleration, the bigger is the DMSA.

### 5.3. Optimal Combined Contour of DMSA Using Both $z_{mag}$ and $z_{absN}$ (or $z_{absE})$

**Figure 14:**
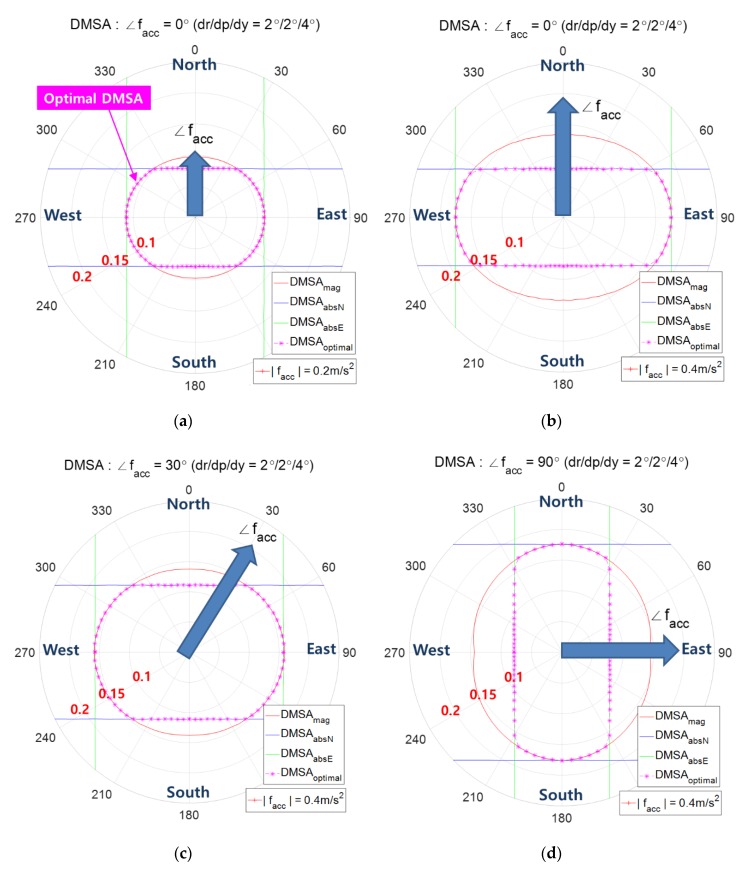
Optimal combined contour (pink color) of DMSA using both $z_{mag}$ and $z_{absN}$, the angle denotes the direction of $f_{s}$, i.e., ∠$f_{s}$; (**a**) $∠f_{acc}=0$ °, |$f_{acc}|=0.2\;m/s^{2}$, (**b**) $∠f_{acc}=0{}^{\circ}$, |$f_{acc}|=0.4\;m/s^{2}$, (**c**) $∠f_{acc}=30{}^{\circ}$, |$f_{acc}|=0.4\;m/s^{2}$, and (**d**) $∠f_{acc}=90{}^{\circ}$, |$f_{acc}|=0.4\;m/s^{2}$.

When both decision variables $z_{mag}$ and $z_{absN}$ (or $z_{absN})$ are used, the optimal combined contour can be obtained by combining Figure 11 and Figure 12. Here, the optimal combined contour, colored in pink, is the inner most combined contour from the two contours of DMSA using $z_{mag}$ and $z_{absN}$. Figure 14a shows the optimal combined contour in the case of |$f_{acc}|=0.2\;m/s^{2}$ and ∠$f_{acc}=0{}^{\circ}$. When the magnitude is increased to |$f_{acc}|=0.4\;m/s^{2}$ while maintaining the direction, Figure 14b shows that the threshold $\gamma_{mag}$ is increased, but the threshold $\gamma_{absN}$ does not change. In Figure 14a,b, it is observed that when $f_{acc}$ heads north, as |$f_{acc}|$ increases, the difference $\gamma_{mag}$-$\gamma_{absN}$ becomes larger and the range of angles where $DMSA_{absN}<DMSA_{mag}$ holds, becomes larger. Figure 14c,d show the case of $∠f_{acc}=30$° and $∠f_{acc}=90$°, respectively. When $f_{acc}$ heads east, i.e., $∠f_{acc}=90$° as in Figure 14d, $z_{absE}$ and $\gamma_{absN}$ should be used instead of $z_{absN}$ and $\gamma_{absN}.$

**Figure 15:**
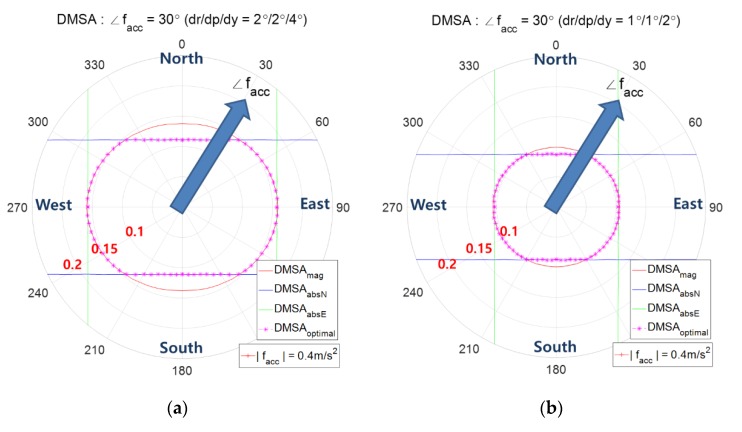
Combined Contour of DMSA with respect to the AHRS attitude accuracy.

Figure 15 shows the combined contour of DMSA using both $z_{mag}$ and $z_{absN}$ according to the AHRS attitude accuracy. It shows that as the attitude accuracy enhances, the combined contour of DMSA shrinks.

**Figure 16:**
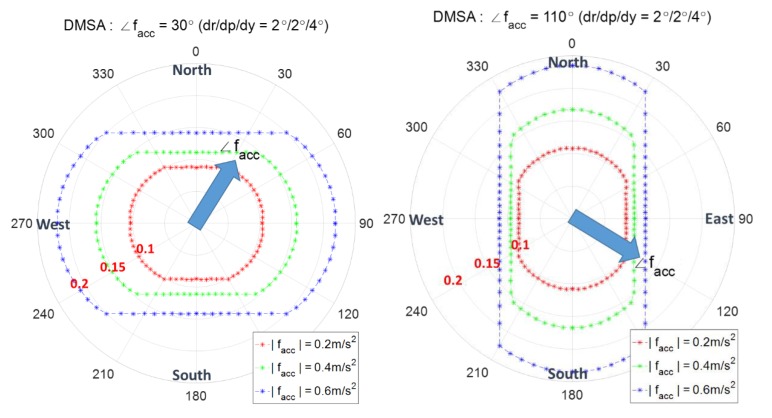
Combined contour of DMSA with respect to the magnitude of $f_{acc}$.

Figure 16a,b show the case of $∠f_{acc}=30$° and $∠f_{acc}=120$°, respectively, with |$f_{acc}|=0.2,\;0.4,0.6\;m/s^{2}$. The decision variable $z_{absN}$ is used for $∠f_{acc}=30$° and $z_{absE}$ is used for $∠f_{acc}=120$°. It is observed that as |$f_{acc}|$ increases, the range of angles using $z_{absN}$ (or $z_{absE}$) becomes larger.

**Figure 17:**
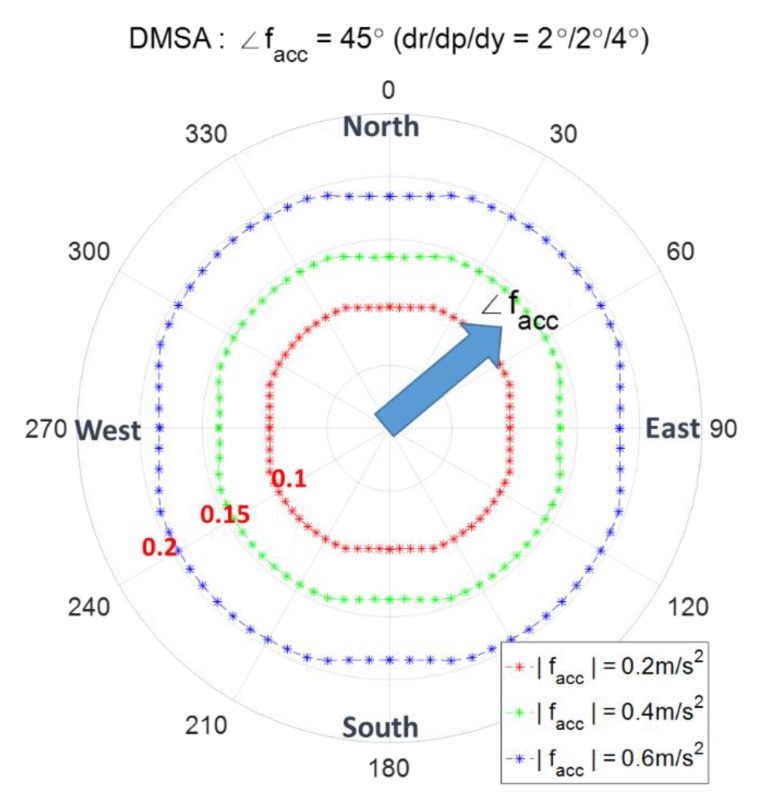
Combined contour of DMSA with respect to the magnitude of $∠f_{acc}=45$°.

Figure 17 shows the case of $∠f_{acc}=45$° and the decision variables $z_{mag},\;z_{absN}$ and $z_{absE}$ are all necessary to obtain the optimal combined contour.

## 6. Conclusions

In this study, a direct GPS spoofing detection method is proposed, with AHRS and accelerometers via the difference of the acceleration estimated from GPS receiver and the acceleration measured from IMU. From the acceleration error expressed in the navigation frame, two decision variables are defined for spoofing detection. One decision variable $z_{mag},$ which may be commonly used, is defined as the magnitude of the horizontal acceleration error. The other decision variable $z_{absN}$ (or $z_{absE}$) is defined as the magnitude of the north (or east) component of the acceleration error.

The spoofing detection performance can be evaluated using the detection probability, which can be calculated from the probability density function of both decision variables. The decision variable $z_{absN}$ shows higher detection probability than $z_{mag}$ in the condition that both moving acceleration and spoofing acceleration are heading within roughly 25° from the north or south. Similarly, the decision variable $z_{absE}$ shows higher detection probability than $z_{mag}$ in the condition that both moving acceleration and spoofing acceleration are heading within roughly 25° from the east or west.

When detectable minimum spoofing acceleration (DMSA) is used, the decision variable $z_{absN}$ (or $z_{absE}$) shows smaller DMSA than $z_{mag}$ in the condition that both moving acceleration and spoofing acceleration head are within roughly 25° from the north–south direction (or east–west direction).

The spoofing acceleration can happen to be any direction. Thus, given a pre-defined false alarm probability, the best algorithm to detect GPS spoofing is that the three decision variables $z_{mag},\;z_{absN},$ and $z_{absE}$ are calculated and compared with the corresponding threshold, and declare the existence of the GPS spoofing if any of the three decision variables exceed the corresponding threshold.

The proposed GPS spoofing detection method in this paper depends on the acceleration error. If a ground vehicle runs across road irregularities such as potholes, bumps, and rubble, etc., then accelerometers may show large changes and deteriorate the GPS spoofing detection performance. Therefore, the flying or driving environment may have an effect on the GPS detection performance.
